# DEEP PROTEOMIC PROFILING OF ACCELERATED KIDNEY AGING AND SENESCENCE BURDEN IN MACACA MULATTA

**DOI:** 10.1093/geroni/igae098.1168

**Published:** 2024-12-31

**Authors:** Christina King, Jordan Burton, George Schaaf, Stephen Kritchevsky, J Cline, Jamie Justice, Ellen Quillen, Birgit Schilling

**Affiliations:** Buck Institute for Research on Aging, Novato, California, United States; Buck Institute for Research on Aging, Novato, California, United States; Wake Forest University School of Medicine, Winston-Salem, North Carolina, United States; Wake Forest University School of Medicine, Wake Forest University School of Medicine, North Carolina, United States; Wake Forest University School of Medicine, Winston-Salem, North Carolina, United States; XPRIZE Foundation, Winston-Salem, North Carolina, United States; Wake Forest University School of Medicine, Winston-Salem, North Carolina, United States; Buck Institute for Research on Aging, Novato, California, United States

## Abstract

Whole-body ionizing radiation (IR) is a stressor that causes damage to DNA, lipids, and proteins, and in the latent-phase, may lead to oxidative stress, inflammation, and cellular senescence. Slowly-reproducing tissues, including kidney, are more likely to react to DNA damaging stressors, which may exacerbate aging processes. Quantitative proteomics was employed to gain molecular insights into how IR exposure may drive renal aging processes. For this analysis, kidney cortex tissues (N=38) were obtained from the Wake Forest Non-Human Primate Radiation Late Effects Cohort. Protein lysates (100 µg) were digested and peptides were desalted. Peptides were analyzed on a timsTOF HT mass spectrometer (Bruker), raw data was analyzed using protein-analysis software, and significantly-altered proteins were subjected to bioinformatic analysis. 5,472 protein groups with at least two unique peptides were identified in the kidney cortex. The influence of 1) kidney disease (KD) in irradiated individuals, 2) only kidney disease, and 3) only irradiation was studied. Interestingly, over 75% of significantly-altered proteins are significantly upregulated. This includes extracellular matrix proteins tenascin and periostin, which are potential aging biomarkers. Upon mapping this dataset to homologous human proteins, proteins associated with kidney disease among irradiated animals are related to EGF pathways, integrin signaling, and fibrosis. Significantly-altered proteins related with kidney disease, independent of irradiation, are related to EGF pathways, integrins, and fibrosis whereas significantly-altered proteins related to irradiation exposure are involved in fibrosis. Similarities in biological processes from significantly-altered proteins in either kidney disease or irradiation highlight a potential synergistic effect of irradiation on kidney disease.

